# Constructing the program theory: an implementation science approach to understanding a successful interdisciplinary team-based model of rheumatology care

**DOI:** 10.1186/s43058-026-00870-w

**Published:** 2026-02-06

**Authors:** Lauren K. King, Daphne To, Zeenat Ladak, Laura Oliva, Carrie Barnes, Catherine Hofstetter, Diane Tin, Carter Thorne, Noah Ivers, Jessica Widdifield, Celia Laur, the Canadian Rheumatology Implementation Science Team (CAN-RIST)

**Affiliations:** 1 Department of Medicine, Temerty Faculty of Medicine, University of Toronto, Toronto, Canada; 2 Institute of Health Policy, Management, and Evaluation, University of Toronto, Toronto, Canada; 3 Applied Psychology & Human Development, University of Toronto, Toronto, Canada; 4 Women’s College Hospital Institute for Health System Solutions and Virtual Care, Toronto, Canada; 5 Patient Partner, Barrie, Canada; 6 Patient Partner, Toronto, Canada; 7 Li Ka Shing Research Institute, St. Michael’s Hospital, Toronto, Canada; 8 Centre of Arthritis Excellence, Newmarket, Canada; 9 Department of Family & Community Medicine, Temerty Faculty of Medicine, University of Toronto, Toronto, Canada; 10 Dalla Lana School of Public Health, University of Toronto, Toronto, Canada; 11 Division of Rheumatology, Li Ka Shing Research Institute, St. Michael’s Hospital, 30 Bond Street, Room 3-057, Toronto, ON M5B 1W8 Canada; 12 ICES, Toronto, Canada; 13 Sunnybrook Research Institute, North York, Canada

**Keywords:** Rheumatology, Models of Care, Integrated Care, Team-Based Care, Implementation Science, Diffusion of Innovation

## Abstract

**Background:**

Team-based rheumatology care, with rheumatologists and interdisciplinary health professionals (IHPs) working collaboratively, is a promising solution to improve service capacity and patient outcomes. However, increasing the number of team members does not mean a team successfully improves care quality. We sought to identify the key ingredients of a successful team-based rheumatology model to inform spread and scale of effective team-based rheumatology care.

**Methods:**

Informed by implementation science frameworks, we used a case study approach to construct the program theory of a leading example of team-based rheumatology care in Ontario, Canada. We completed semi-structured interviews (patients [*n* = 15], health professionals [*n *= 11]), naturalistic observations (*n* = 3), and document reviews. We conducted framework analysis and iteratively developed an Implementation Research Logic Model, linking determinants of optimal team-based rheumatology care to implementation strategies, mechanisms of action, and outcomes.

**Results:**

Diverse skill sets of team members enabled comprehensive, person-centered care. IHPs assumed expanded responsibilities, engaging in all aspects of rheumatology care, increasing care capacity and timely access. Training and mentorship were essential for IHP skill development to implement expanded responsibilities at the highest professional scope. Continuous evaluation and adaptations of the model were essential to address evolving care needs. Stable funding was critical for initiation and sustainability.

**Conclusion:**

Successful team-based rheumatology care involves a patient-centered, adaptable care model supported by sustainable funding, skilled workforce, strong leadership and continuous evaluation. By identifying key components and understanding how they achieve their impact, we have gained valuable insights to inform implementation, spread, and scale of such models.

**Supplementary Information:**

The online version contains supplementary material available at 10.1186/s43058-026-00870-w.

Contributions to the literature

- This is the first study to evaluate and articulate the program theory behind a successful interdisciplinary team-based model of rheumatology care, providing insight into how such a model works and achieves its outcomes.
- We found that successful team-based rheumatology care involves a patient-centered, adaptable care model supported by sustainable funding, skilled workforce, strong leadership, effective communication, and continuous evaluation. Implementation strategies such as ongoing training, mentorship, standardized assessments, and infrastructure optimization fostered workforce development and interdisciplinary collaboration, ultimately leading to improved care access, equity, patient satisfaction, and perceived enhancements in disease management.
- This study also demonstrates how implementation science frameworks can be applied within rheumatology research to better understand and explain the functioning of complex health interventions.
- Our study provides real-word learnings that support future spread and scale of interdisciplinary rheumatology care by offering a theoretical foundation and practical understanding of innovation components and implementation strategies that may underlie effective team models.

## Introduction

Worldwide, rheumatology workforces are facing a rising burden of rheumatic and musculoskeletal disorders (RMDs)[1–3], which include inflammatory, non-inflammatory, and autoimmune conditions (e.g., rheumatoid arthritis, psoriatic arthritis and osteoarthritis) [4]. Rheumatology wait times are among the longest of all specialist physicians in Canada [5] and remain an issue globally [6]. Further, individuals with RMDs have increasingly complex care needs [7, 8], and encounter significant challenges in accessing ongoing comprehensive, integrative care throughout their disease journey [9, 10]. Innovative healthcare solutions are needed to support and enhance rheumatology care delivery and keep pace with growing patient demands.

Interdisciplinary team-based models of rheumatology care are a promising answer to improve access to patient-centered care for people living with RMDs [11]. In an interdisciplinary rheumatology team, rheumatologist(s) and health professional(s), from different disciplines with complementary backgrounds and skills, integrate their expertise to collaboratively assess and manage patient care needs [12]. Unlike multidisciplinary models of rheumatology care, where providers work independently in a sequential manner within their specific roles (e.g. rheumatologists focus on diagnosis and pharmacologic management and physiotherapists address rehabilitation), interdisciplinary health professionals (IHPs) – which may include physiotherapists, occupational therapists, nurses, pharmacists – collaborate in decision-making with rheumatologists and assume responsibilities beyond their traditional practice scope [13]. Intuitively, integrating IHPs into rheumatology settings within an interdisciplinary model may enhance care capacity by expanding the pool of rheumatology health professionals to increase service availability and efficiency. The diversity of expertise within interdisciplinary team-based models of rheumatology care may also improve patient outcomes [14, 15] and patient satisfaction with care [16, 17]. As such, the Canadian Rheumatology Association recommends incorporating IHPs within rheumatology practice to promote and enhance workforce capacity [18].

Despite the potential for addressing the healthcare needs for people living with RMDs, interdisciplinary team-based models have not been widely implemented. This lack of implementation may be explained, in part, by the limited understanding of how these models operate, what makes a successful interdisciplinary rheumatology team, and how the model achieves its effects. Adding team members, without clarification of roles and without an overarching approach to patient-centred collaboration, will not increase capacity to effectively address RMDs. Thus, improved understanding how team-based rheumatology models are best implemented is crucial to develop a plan for spread (moving from one location to the next) and scale (developing the infrastructure to underpin and support widespread implementation) [19].

Our objective was to elucidate the “program theory” underlying an exemplar case of team-based rheumatology care. A program theory describes how an intervention is expected to lead to its effects and under what conditions [20]. Program theories are typically not well developed for complex, real-word interventions. By deeply exploring an effective team-based rheumatology model [21, 22] and describing its program theory, our primary goal was to contribute to the key knowledge required to successfully spread, scale, and sustain such models of rheumatology care to improve care for people with RMDs. We further illustrate how implementation science frameworks can be used within a case study to help understand and communicate the functioning, mechanisms of action, and effects of complex interventions within real-world rheumatology settings.

## Methods

### Design

We used case-study methodology [23] to construct the program theory of an exemplar case of team-based rheumatology care and develop an Implementation Research Logic Model (IRLM), as described by Czosnek et al*.* [24]. The IRLM provides a structured way to integrate core elements of implementation science, making it easier to visualize and communicate the relationship between an intervention, implementation strategies used, and outcomes [25]. Informed by implementation science frameworks, we conducted an in-depth exploration within the case’s real-life context [26] to construct “how”, “what”, and “why” the team-based model achieves its effects, and to explore presumed causal links and pathways [23]. We approached this research with an interpretivist stance, maintaining a focus on participants' meanings and experiences within their complex social context [27].

We reported this study according to the Consolidated Criteria for Reporting Qualitative Research (COREQ) checklist (Additional File 1). This study was approved by the University of Toronto Health Sciences Research Ethics Board (REB# 45644).

### Setting

The health care system in Ontario is publicly funded (through taxes). Typically, most physician services, including rheumatologist visits, are covered by the publicly funded insurance. Patients need a referral from a primary care provider or other specialist to see a rheumatologist. Most rheumatologists practice independently in outpatient settings and receive reimbursement from the Ontario Health Insurance Plan via fee-for-service payments for the services they provide. In the outpatient setting, services by non-physician health professionals are generally not covered by the Ontario Health Insurance Plan and must be paid for directly by the patient or through private insurance, with partial exceptions for individuals on social assistance or those over the age of 65. In the community rheumatology outpatient setting, there is no provincial funding for IHPs. The one exception, to our knowledge, is the Centre of Arthritis Excellence (CArE), the focus of this case study.

### Case description

Located in Newmarket, Ontario (semi-urban city north of Toronto), CArE is the province’s only government-funded, community-based interdisciplinary rheumatology care team-based model. Established in 1991 as The Arthritis Program, it was initially funded by a grant from the Ontario Ministry of Health, administered by a regional hospital, and subsequently subsumed by the hospital global budget. In 2021, funding transitioned to the Ontario Ministry of Health. The funding model is based on the Ontario Family Health Team funding model, a type of alternative payment plan which includes a bundled payment contract to cover the human resources to support the model (IHP salaries), administration, and other operating expenses. Rheumatologists’ services at CArE are remunerated fee-for-service. In non-integrated care models in the community, rheumatologists’ services are also remunerated fee-for-service, however they are responsible for operational expenses (e.g., space, personnel).

CArE characterizes itself as “a portal of care for musculoskeletal services in the community; accessible to all primary care providers, rheumatologists, other subspecialties and interdisciplinary health providers”. Within the umbrella of services provided, this includes a community-based interdisciplinary rheumatology health team (rheumatologists working collaboratively with IHPs that have included pharmacists, occupational therapists, physical therapists, nurses, kinesiologists, dietitians, and social workers), disease-specific educational programs (for inflammatory arthritis, osteoarthritis, fibromyalgia, and osteoporosis), and individualized services rendered solely by IHPs if rheumatologist consultation is not needed (e.g. physical therapy is provided by physiotherapists for a patient with an acute regional musculoskeletal condition). As services are tailored to individual needs, every patient does not need to see every type of IHP, not all the IHPs participate in shared patient and team decision-making at the same time, and not every patient will require consultation or review by the rheumatologist. This study, however, focuses on the interdisciplinary rheumatology team model, where patients with chronic RMDs (e.g., with inflammatory arthritis) are longitudinally evaluated and managed collaboratively by rheumatologists and IHPs. Evidence suggests that CArE services a substantially higher volume of patients with RMDs, and enhances patient outcomes compared to conventional rheumatology practices [21, 22].

### Research approach

The updated Medical Research Council (MRC) Framework for Developing and Evaluating Complex Interventions [20] guided our overall methodological approach. The updated MRC framework emphasizes developing an understanding of the active components of the innovation and how they interact with one another and their context to generate intended and unintended outcomes [28].

To develop the program theory at CArE, we drew on two implementation frameworks and one taxonomy, which we applied to data collection and analysis. The *Consolidated Framework for Implementation Research (CFIR) 2.0* [29] was used as a determinant framework to describe the barriers and enablers to optimal rheumatology care at the innovation, outer setting, inner setting, individual, and implementation process levels [29]. For this study, optimal rheumatology care was defined as care that is accessible, evidence-based, outcome-driven, and person-centered. The *Expert Recommendations for Implementing Change (ERIC)* [30] taxonomy outlines 73 distinct implementation strategies, defined as “methods or techniques used to enhance the adoption, implementation, and sustainability of a clinical program or practice” [31]. These implementation strategies have been grouped into ERIC strategy clusters [32] that were applied to understand and describe the implementation strategies at CArE. The *Implementation Outcomes Framework (IOF)* [33] was used to understand and describe implementation outcomes, focusing on five key areas: acceptability (the extent to which the care model is accepted by patients, health professionals, and staff), appropriateness (the perceived fit, relevance, or compatibility of the intervention for the setting, provider, or patient population), fidelity (adherence to the vision of providing interdisciplinary, comprehensive, and accessible rheumatology care), penetration (the model’s ability to deliver equitable care and reach all appropriate patients with RMDs), and sustainability (the potential for this care model to continue in the long term). We also sought to understand and describe relevant service outcomes [34] and patient outcomes as specified by Proctor et al*.*[35].

We used the IRLM [25] as the program logic to link the above frameworks and organize the implementation components. The IRLM specifies key implementation components (defines the intervention being implemented in the specific context and implementation strategies used to function), maps implementation determinants and mechanisms (linking barriers and enablers to implementation strategies and how these factors influence implementation processes and outcomes), and aligns implementation strategies and outcomes (connecting implementation efforts to measurable short-term and long-term implementation, service, patient outcomes).

### Data collection

**Fig. 1:**
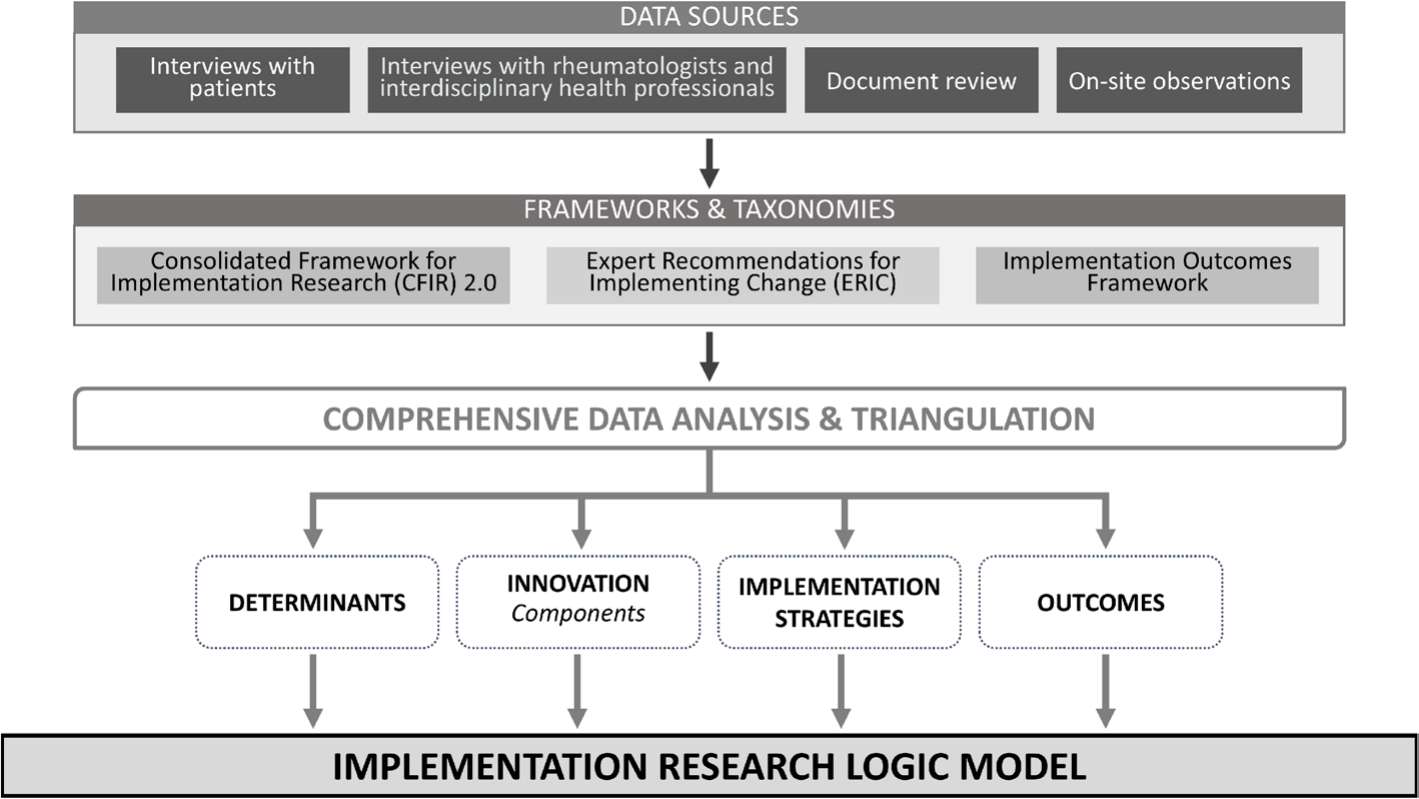
Research Approach Overview: Multi-source data organized using implementation science frameworks and relevant service and patient outcomes

We collected data from multiple sources to allow for triangulation [23, 26]. An overview of our research approach is presented in Fig. 1.

#### Semi-structured interviews with health professionals and patients

Two doctoral trainees with prior qualitative research experience conducted one-on-one interviews (lasting 30–45 min) via telephone or video with CArE patients (ZL) and health professionals (DTo). To be eligible, study participants must have received care or provided healthcare services at CArE over the past 10 years (i.e., between 2014 and 2024). We used purposive sampling to incorporate a wide range of perspectives from information-rich participants exposed to the team-based care model. Interview guides, specific to each patient and provider group (Additional Files 2, 3), comprised open-ended questions followed by probing questions informed by CFIR 2.0 [29], ERIC [30], and IOF [33] to capture key determinants, intervention components, mechanisms of action, and their links to outcomes (implementation, service, patient). The interview guides also sought to identify key factors related to the patient journey to enable us to comprehensively communicate the CArE exemplar, including content, procedures, and activities. We collected demographic characteristics at the end of each interview. Recruitment ended when the analytic team determined by consensus that additional interviews for each participant group provided confirmatory data but did not expand developing analytic categories. Interviews were audio recorded and professionally transcribed. Interviewers had no prior relationship with participants. We did not employ member checking.

#### Naturalistic observations

Between March and May 2024, three researchers (both doctoral trainees, DTo and ZL, as well as LK, a rheumatologist and scientist) attended the clinic on separate days (4 h per visit; 12 h total) to observe and document aspects of the model to gain an understanding of how the clinic operates, roles, tasks and functions of clinic staff, physical space, health information system, and the interactions between interdisciplinary team members. Observers did not have prior relationships with anyone observed. Narrative field notes were recorded during and immediately after each visit and observers discussed observations between visits. Multiple observers, with different backgrounds and levels of experience, enhanced the credibility of results through triangulation [26, 36]. Observations were guided by an observation tool (Additional File 4), informed by the study aims and relevant frameworks (CFIR, ERIC and IOF). Observers asked questions for clarification but did not interrupt clinical care.

#### Document review

We obtained organizational documents from CArE (e.g., reports, strategic plans, patient handouts) and searched publicly available documents online (search in Google for “Centre of Arthritis Excellence,” “The Arthritis Program,” and similar terms) to provide historical and contextual information about CArE.

#### Data analysis

Informed by the IRLM structure, and drawing on two implementation science frameworks and one taxonomy, we deductively coded interview transcripts, observation notes, and organizational documents into broad categories: innovation description, patient journey, determinants of optional rheumatology care based on CFIR 2.0 [29], implementation strategies following clusters of ERIC [30], and outcomes, including IOF [33], and service outcomes [34] and patient outcomes as specified by Proctor et al*.* [35]. To maintain a standardized approach to the data, we used a single codebook for health professional transcripts, observation notes, and organizational documents. A second codebook was used for patient interviews that followed the same structure but with code descriptions adapted to data from patients. Our analytic team comprised of researchers with implementation science and qualitative expertise (LK, CL, DTo, ZL, LO), chiropractic experience (DTo), and rheumatology expertise (LK). Up to three interview transcripts, observations, and documents were coded in duplicate by the analytic team until consensus was achieved on application of codes after which the remainder of transcripts were divided and coded independently, in duplicate. Each pair of coders applied codes independently and any discrepancies were resolved in a face-to-face consensus meeting.

We used the framework method to categorize, organize, and analyze the data [37]. This method shares similarities with thematic analysis as an approach to identify commonalities and differences in qualitative data, while drawing descriptive and/or explanatory conclusions clustered around themes, and reconciling the perspectives within different collected data sources [37]. Data was triangulated across sources [23, 26] that provided distinct insights. Each data source was coded separately, with interviews providing more nuanced perspectives, while observations and documents filled gaps regarding structure and function of the clinic. Data for each code was compared and synthesized through a structured process to identify areas of corroboration, complementarity as well as divergence across data sources. The analytic team discussed patterns across the data and explored alternate interpretations. We also engaged in reflexive research practices by critically examining and challenging our own assumptions and positionality. We organized transcribed interviews, observations, and document data using NVivo 15 (QSR International Pty Ltd).

All results were integrated to inform the program theory, depicted using an IRLM [38].

## Results

### Data sources

Multiple data sources informed our analyses: *n* = 11 health professional interview transcripts, *n* = 15 patient interview transcripts, *n* = 32 documents, and *n* = 3 observation reflective transcripts (from 12 observational hours). We present characteristics of study participants and data sources in Table 1.

**Table 1:** Characteristics of Study Participants and Data sources

PATIENT Participants	TOTAL *n* = 15
Age in years – Median [range]	62 (42–78)
Sex – n (%)	
Male	8 (53%)
Female	7 (47%)
Race/Ethnicity – White, n (%)	14 (93%)
Diagnosis – n (%)	
Rheumatoid Arthritis	6 (40%)
Spondylarthritis (PsA, AS)	4 (27%)
Osteoarthritis	2 (13%)
Other inflammatory rheumatic diseases (CTD, PMR, SLE)	3 (20%)
Employed – n (%)	
Yes	5 (33%)
No	5 (33%)
Retired	5 (33%)
HEALTH PROFESSIONAL Participants	TOTAL *n* = 11
Age ≥ 50 years – n (%)	5 (45%)
Sex – n (%)	
Male	2 (18%)
Female	9 (82%)
Profession, n	
Rheumatologist	3 (27%)
Interdisciplinary health professional*	8 (73%)
> 2 years of experience at CArE – n (%)	5 (45%)
≥ 10 years in professional practice – n (%)	9 (82%)
ON-SITE CLINIC OBSERVATIONS (4 h each)	TOTAL *n* = 3 (12 h)
DOCUMENT ANALYSIS	TOTAL *n* = 32
Educational material	
Patients	4
Health Professionals	7
Strategic	5
Promotional	
Educational program information	7
Patient testimonials	5
About CArE	2
Academic publications	2

*AS* Ankylosing spondylitis*, CArE* Centre of Arthritis Excellence*, CTD* Connective Tissue Disease*, PMR* Polymyalgia rheumatica*, PsA* Psoriatic Arthritis*, SLE* Systemic Lupus Erythematosus

^*^Disciplines of health professionals not further indicated to maintain confidentiality of participants. Some health professionals also had leadership and administrative roles in addition to their role in clinical care

### Innovation components

The CArE model is designed as a fully integrated, team-based approach to rheumatology care. Rheumatologists and IHPs collaborate and share responsibilities within the same physical space to deliver rheumatology care with IHPs involved in the entire patient care continuum. The clinic space and shared electronic medical record accommodates multiple team members, with dedicated space for meeting of additional care needs (educational sessions, splinting, and rehabilitation space).

A standardized patient referral form enables IHPs to triage patients based on diagnosis, urgency, and care needs, ensuring they are directed to the most appropriate and efficient care pathway. Priority is given to individuals with potential inflammatory rheumatic disease for the earliest possible evaluation, with IHPs (physical therapists and occupational therapists) responsible for acquiring supporting documentation of additional relevant investigations/reports (if needed), and patient scheduling. The initial patient intake assessment (history, physical exam, review of investigations) is completed by a physical therapist or occupational therapist, who is taking on greater responsibility for care than usual for their professional role (practicing at the highest scope of their professional designation). The patient is then seen and examined by a rheumatologist. The IHP(s) and rheumatologist, together with the patient, collaboratively construct a comprehensive management plan which might draw on the expertise and services of additional members of the interdisciplinary team (e.g., pharmacist or occupational therapist) dependent upon patient needs. Both rheumatologist and IHP(s) share in engaging/educating patients on the care management plan, which may include pharmaceutical and/or non-pharmaceutical interventions, and integrates and focuses on prevention, early intervention, continuous monitoring (e.g. to prevent complications, minimize treatment side effects), and how to access team services between follow-up visits as patient needs evolve. Follow-up visits are completed in a similar manner. Between scheduled visits, queries from patients, typically received as a phone call, are returned by the most appropriate team member (e.g., medication questions returned by the pharmacist). Patients and team members complete standardized assessment forms at each visit to track outcomes such as pain, function, and disease activity, which are electronically monitored over time through the electronic medical record system. Documentation is shared across the team with the IHP(s) sharing responsibility in diagnostic/laboratory testing requisitioning, providing consultation reports to referring physicians, and other form completion (such as prior authorization forms for drug insurance approval, disability and medical leave forms). IHPs deliver additional formal disease education sessions (in person and virtual) on topics such as “self-management of inflammatory arthritis” on a rotating basis. Team members communication informally within shared workspaces and formally through shared electronic medical record.

### Determinants

We identified multiple determinants of optimal rheumatology care (CFIR 2.0 domains in *italics*). We found that key enablers included a model of care provision focused on providing timely, evidence-based, adaptable, outcome-driven, and patient-centered and equitable care (*innovation*). Enablers influenced by the health system/community (*outer context*) include sustainable funding, networks of referring providers (usually primary care) directing patients to rheumatology care in a timely manner, and workforce availability of skilled health professionals to deliver arthritis care. Within the clinic space (*inner setting*), enablers include sufficient physical space to deliver comprehensive care, strong leadership, and robust avenues for communication within the clinic space. Individual attributes include patient self-management skills and health professionals’ expertise (*individual*). Additional characteristics of health professionals include motivation, a desire for interdisciplinary collaboration and a commitment to building high-quality, trust-based relationships within the team. Finally, a dynamic *implementation process* ensures care delivery adapts to contextual, provider, and patient needs through continuous evaluation methods to monitor progress. Overall, the interplay of these determinants facilitates the delivery of optimal rheumatology care. We present descriptions of key determinants of optimal rheumatology care with illustrative quotes, organized according to CFIR 2.0, in Table 2.

**Table 2:** Determinants of optimal rheumatology care by domain of the Consolidated Framework for Implementation Research 2.0

CFIR Domain, constructs and description	Illustrative Quote
**Innovation: Components of optimal rheumatology care** *Innovation relative advantage* *Innovation adaptability*	
*Timely* care is provided from initial consult to ongoing follow-up	*“Our goal—it’s to improve access to care for patients, so that patients don’t have to wait as long to get a diagnosis. We can get them diagnosed and started on appropriate treatment earlier without the wait, because we know that, you know, inflammatory arthritis situation, getting them treated early is better for their outcomes. So that’s a big goal is getting them the appropriate care at the appropriate time, by the appropriate people.”* HP10
*Evidenced-based care* is accessible and comprehensive within one program	*“The patient gets the advantage of a therapist who can talk to them and make sure they understand what they've got to do. … explaining things in a little more detail, and setting up all those appointments. And they get to talk to the pharmacist about their meds sometimes before they walk out the door, and other times with a setup, either phone or in-person visit, to review their medication needs. And that just doesn't happen [at] other places.”* HP10
*Adaptable* model of care is provided that is: • Specific to patient needs • Tailored to patient preferences and needs by providing care in person and virtually • Refined to meet the context/workforce • Flexible to allow different team members to share roles	*“I think it’s [the program] kind of just evolved. That it’s sort of this well-oiled machine that we can sort of like bend, flex, adapt as needed to sort of like make the clinic run smoothly because we all kind of – you know. We all kind of take initiatives as needed to do that.”* HP11
*Outcome-driven* care is provided	*“We give them all the Health Assessment Questionnaire. And then they also get a subjective – like there’s a one-sheet … that basically has kind of a wheel of the things that are their major concerns. And around the wheel it might be pain, fatigue, sleep and then, you know, mobility – and this is on a wheel. And then underneath it says, you know, “Strategies I’m currently using to manage,” and then it sort of lists and then they are able to say what their main concerns are. So that kind of gives us a bit of insight into what’s important to them.”* HP11
*Patient-centered and equitable care* is provided	*“It’s just kind of an understanding of what you're going through. Things to keep in mind in terms of lifestyle, with the pharmacist being able to talk about blood work and what should I be looking for.” Patient14* *“I don’t think rheumatology would be well managed with one clinician. Because it really is a team – I think a team-based model like should be sort of the gold standard because really, it’s sort of addressing all aspects of the patient’s, you know, goals to return to just as normal life as possible.”* HP11
**Outer setting: Health System/Community** *Financing* *Partnerships & Connections* *Local conditions*	
*Stable funding* for team-based care is required to support delivery	*“I will say over and over again that competitive compensation is a challenge.”* HP6
*Referral networks* are needed so healthcare professionals are aware and can make timely and appropriate referrals	*“I think that it has to filter down to obviously, the medical community so that doctors are aware. Also, that people in the community are aware because if their doctors aren't aware of the program or perhaps they're too busy to do a referral or whatever.”* Patient31
*Work force availability,* including workforce training needs	“*Certainly we all like what we're doing. We're making a difference, but at the end of the day, if we can't recruit people, you’re not going to have a good team.”* HP10
**Inner setting: Clinic Space** *Structural characteristics* *Relational connections* *Available resources*	
*Physical space* that facilitates diverse care needs, including infrastructure that supports relevant information technology, and supports optimal organization of clinic tasks and responsibilities	Clinic Observation: [The clinic] has an appearance of a modern medical office. There are multiple clinic rooms and working spaces in addition to a staff bathroom, office area at the back with open cubicles, a closed office for the executive chair, a counseling room that has been turned into a lunchroom, and a meeting room that is used for educational programs. The meeting room has posters from the [program name] on the wall and there's exercise equipment like exercise balls and resistance bands throughout the room. There is large monitor that enables video conferencing
*Leadership* is strong and can meet the needs of the care team	*“I think there almost needs to be two types of leaders… you need that financial and dealing with the Ministry and the political things and – you know, hiring and firing and not that hopefully, I mean no one's getting fired, but dealing with – and even just the management of the computer systems and the – like the heat and the hydro … And then you also need the strong clinical leadership as well, that is also going to help with training and account keeping. You know, figuring out and dealing with the flow of the clinic, and efficiencies and statistics and studies.”* HP10
*Avenues for team communications* are available that support learning and collaboration	*“I think that really – the joint space really adds to learning and collaboration.”* HP2
**Individual: Patients and health professionals** *Innovation recipients* *Innovation deliverers*	
*Patients:* knowledge, skills in self-management, agency and self-efficacy	*“I think just having that additional support, rather than just being on your own, looking at websites like Mayo Clinic or whatever, which you know, sometimes there’s disadvantages to doing all of this, you know, the reference is often Dr. Google, right? Like there could be a disadvantage sometimes to just reading things on the web. So it’s just like having that additional support as I found helpful.”* Patient14 *“It’s a process. Things take time. But we we’re willing to work with it to get it to – because it’s my own body. I have to look after myself.”* Patient18
*Rheumatologist/IHP:* motivation, optimism, knowledge of rheumatic disease, skills in rheumatology care delivery, and high-quality relationships within the team	*“You need good health care professionals. You need people that come in with a diverse set of skills beyond just their health care professionals. And some, you know, even just life skills is helpful. I think when you get a new grad, I think in this type of environment, that would be difficult. I think you need to get someone who’s got a little bit of experience under their belt before they jump into a specialty team. Just so that they can help the patients navigate the system. And if they haven't been a part of that system, it’s hard to navigate the system.”* HP10 *“Having physicians who buy into the model too, right? Like, you can't do this without the physicians who, you know, if they really want Allied Health Team members to be knowledgeable – that they can trust their assessments, they also have to spend time working with people and learning, and teaching, right?”* HP2
**Implementation process: Act of putting innovation into practice** *Teaming* *Assessing needs* *Tailoring strategies* *Adapting*	
Adaptable to diverse patient needs, shifting demands to optimize delivery, including the: • intentional coordination of task related to care delivery • collection of information about priorities and preferences of staff and patients to facilitate optimal care delivery • operationalization of care delivery strategies to fit changing context • modification of the program as needed for optimal care delivery	*“You need a supportive management. You need doctors who are willing to work in a team environment, and who are team oriented as well.”* HCP10 “*When you’re starting a* [interdisciplinary team-based] *program, there’s a lot of patients, right, that you have to have because it’s a new team. Not everything’s going to work well right away. There’s a lot of troubleshooting you have to do.”* HCP1

*IHP* Interdisciplinary Healthcare Professional

### Implementation strategies

We identified many implementation strategies at CArE (ERIC implementation clusters in *italics*). These strategies involve tailored, structured approaches to optimize care delivery that were organically implemented and evolved over time to support the sustainability of the program. Ongoing informal mentoring and formal educational opportunities for IHPs (*Train and Educate IHPs*) support continuous skill development and preparedness for delivering complex rheumatology care. Formative assessments support IHP team development including regular physical exam “calibration” events and educational sessions. This training and education facilitate interdisciplinary, trust, collaboration and sharing of responsibilities. A culture of teamwork supports IHPs functioning in advanced practice roles, maximizing their knowledge and skills (*Support Clinicians*). A champion helped reinforce key messages about the model, supported peers in adopting new processes, and liaised with an executive board to address operational barriers and facilitate ongoing functioning of the team (*Develop Interest Holder Interrelationships*). Patient and family feedback is systematically incorporated into the program delivery to align care with their needs and preferences (*Engage Consumers)*. Standardized patient assessments provide a common language among team members to communicate effectively about patient outcomes (*Evaluative and Iterative Strategies)*. Strategies also include audit and feedback mechanisms that rely on collecting and utilizing formal and informal feedback to help refine service components and delivery and support continuous quality improvement. Tailoring strategies to adapt education, services, and delivery methods, including virtual care, to meet changing care needs (*Adapt and Tailor to Context*). Infrastructure changes, including optimized clinical spaces, create space conducive to care team needs and enhanced communication, including informal real-time communication among team members (*Change Infrastructure*). A stable funding mechanism supports the initiation and sustainability of the model of care, including the functioning of the model through support for the clinic infrastructure and IHP salaries (*Utilize Financial Strategies*). This funding is critical to initiation and sustainment of the model. We present key implementation strategies with illustrative quotes in Table 3.

**Table 3:** Implementation strategies identified, by Expert Recommendations for Implementation Change (ERIC) cluster, with descriptive examples

Implementation Strategy (*sub-strategy* & description)	Illustrative Quote
**Train and educate IHPs: Mentorship and training of IHPs in rheumatology knowledge and skills**	
*Conduct ongoing training* by supporting IHP initial and continuing education, including physical exam education sessions for team	*“They have – because they have those calibration days where they – we all examine the joints and kind of make sure we’re all on the same page and we bring a couple patients in. And it’s not like a scheduled clinic time. It’s like scheduled education time together.”* HCP4
*Provide ongoing consultation,* including formal mentorship to promote competency and confidence in advanced practitioner role	*“We’re a team who’s pretty keen to continue to learn and seek feedback. So I think that environment is a culture that we established early on to say, you know, “What should I have done differently” or, “What did I miss” or, “This is what I think. Can you give me some feedback?”* HCP6
*Shadow other experts* as part of initial training to become familiar with team-based care. Ongoing mentorship to develop skills	“*In the initial training, having that opportunity to shadow somebody who’s doing the other disciplines, then you could learn from them, and so then when something like that comes up for myself in a phone call from a patient, or you know, or during a visit and management of a flair, and I can be confident in my suggestions about, “Maybe you should put ice on this,” or something like that. You know, basic stuff, right? But like, management of a flair, and the messaging around what they should and shouldn't do. So that we're all the same.”* HCP10
*Conduct educational meetings,* including providing the time and opportunity for brief, ongoing educational sessions	*“About once a month we also will have rounds where they may have – they may request a topic that I present on more from a didactic perspective where we do a Q&A session of interesting cases that they’ve had. So we’ve done approach to inflammatory arthritis, to connective tissue disease, to serology, particularly ANA patterns and ENA and that kind of thing. And then we simply read the x-rays.”* HCP1
*Make training dynamic,* including providing ongoing and interactive training that aligns within individual needs of IHPs	*“I mean it definitely was initially like outside of my comfort zone. But I feel with sort of my own independent learning I’ve been doing, and the rheumatologists have been amazing about doing some little educational workshops in the morning where we sort of look at, you know, a case study that’s someone we’ve seen here and then the kind of reviewing like, you know, what would the next steps be.”* HCP11
**Support clinicians: Collaborative culture, support for IHPs working in advanced practice roles**	
*Revise professional roles*, including: - Supporting IHPs to work in an extended scope capacity, taking on added responsibility from traditional roles within their disciplines, including use of medical directives - Fostering the team environment through elimination of hierarchy	*“We try to have our team members work way up to their limit in terms of their scope of practice. So obviously we don’t ask anybody to work outside their scope but we certainly want to push that envelope, right. So most physiotherapists and occupational therapists aren’t doing joint counts and talking about inflammatory arthritis. So we certainly know that that’s kind of pushing them to – I don’t want to say the limit, but certainly pushing them to their scope.”* HCP1 *“I can only speak to what I've experienced, but I don't think that Allied Healthcare providers typically do the kind of stuff that we did in terms of the rheumatology clinic, where we would assess, and then provide a little bit of a snapshot of their issues and what the recommendations are. It’s almost, like, you know, working in a model of resident doctors and doctors.”* HCP2
*Facilitate relay of clinical data* to providers by providing standardized outcome metrics to support communication of disease status across team members	*“But we’re mainly going based on their PR – like their patient-reported outcomes and the HAQ* [Health Assessment Questionnaire]*”* HCP11
**Develop interest holder interrelationships: Program champions**	
*Identify and prepare champions,* by having a program champion with ownership and accountability to ensure program provides high quality care and appropriately evolves over time	*“…the leading and also the accountability of having, you know, that program champion take on the leadership role. And because we’re all leaders, at the end of the day you’re in different programs. So I think coming with that lens on moving, you know, changes and innovation forward has helped a lot of having that both accountability as well as the leadership role within the team.” HCP*5
*Involve executive boards,* through a board of directors who oversees and directs activities	*“I mean we have a very eager and experienced board so they’re – we rely on them to some degree to kind of be reaching out to Ministry and partners in the community to help provide some of that support in the future.”* HCP1
*Capture and share local knowledge* to support ongoing learning from interdisciplinary teams	*“As far as team-based models, we’re part of the Association of Family Health Teams – we bring all that back to CARE.”* HCP3
*Obtain formal commitments,* through preparation and submission of proposal levering existing relationship with funders to obtain program funding commitment	“*We had interactions as well with the Ministry of Health. Again, informal, getting mentioned in various white papers that they had. So, these all caused the body of knowledge about our program to be developed and reinforced and endorsed, or whatever the words are…. we made that… submission to the ministry. This was initially passed aside, but [date], I received a call from the minister’s office saying that it was going forward.”* HCP16
**Engage consumers: Formal avenues for patient/family input**	
*Involve patients/consumers and family members,* through the creation of patient family advisory committee facilitates involvement of consumers	“*Over time, we did also develop a patient family advisory committee… They were very instrumental and also important in our submissions to the ministry.”* HCP16
**Use evaluative and iterative strategies: Ongoing program evaluation**	
*Audit and provide feedback,* through: • Ongoing solicitation of formal and informal feedback to plan refinements in program • Formative assessments of team members with feedback	*“We do keep track of the patient flow in terms of when the referral is received, when their initial patient visit is, *etc*. So we do have measures in terms of timeline. One thing that we’re trying to capture more is emergency room avoidance. So we do have some measures looking at that as well. So those are kind of the main ones that we use more and then obviously patient volume, workshop attendance, workshop satisfaction. So we send surveys after each workshop to gauge that. So those are a couple of things.”* HCP1 Clinic Observation: I noticed a poster on one side of the wall with post-it notes for feedback on the “two-step triage process”. It looks like healthcare professionals have been providing feedback on these sticky notes. There were checkmarks on ones that appeared to be addressed
*Purposefully reexamine the implementation* using an iterative approach to continually improve thorough feedback of team members	*“When our team members would say something doesn’t work, they’d have to give that rationale. And then if it didn’t work, we’d change it. Why would we keep it?”* HCP16
*Obtain and use patients/consumers and family feedback* through frequent evaluations (formal and informal) of care provided as part of the program	“S*urveys about the rheumatology clinic itself and how satisfied were people with those kinds of visits and the program itself, like, the inflammatory arthritis stuff. So there were definite attempts to collect information and learn from what patients would want. We also did try stuff like focus groups and that kind of stuff. So a variety of different ways to collect information.”* HCP2
*Develop and implement tools for quality monitoring* by using standardized metrics to ensure patient outcomes are evaluated and addressed	*“We’re asking every time they visit about their pain, their morning stiffness, their sleep. And we also ask them about their general feeling of wellness. So, you know, just their global feeling of how well they’re feeling; zero being really well, 10 being really unwell. So we sort of have that.”* HCP11
**Adapt and tailor to context: Refining and adjusting program delivery to meet changing care needs**	
*Tailor strategies,* including changes in the program over time (changes to education, services offered, training to IHPs) to meet needs of patients and adjusting means of delivery (including use of virtual care)	*“We do want to be wary of maintaining the core elements that make the program successful. So I think we look at the opportunities to add value very carefully.”* HCP1
**Change infrastructure: Clinical space conducive to care team needs**	
*Change physical structure and equipment,* including developing clinical space (clinic rooms, education rooms, electronic medical records) to be conducive to optimal care delivery	*“Luckily we do have a big classroom, so we have like a multiuse space that we call the classroom that’s where the workshops are. And yeah, it was – the furniture in there was purposely picked so that the tables are foldable. The chairs are stackable. So that within actually 10 min we can clear the entire space and use it as a gym.”* HCP1
**Utilize financial strategies: Funding to support model**	
*Fund and contract for the clinical innovation,* including funding to resources program, including space, health professional salaries, and ongoing education of team members	*“We have a lot of support for continuing education, conference attendance.”* HCP3

Specific implementation strategies from the ERIC are provided in italics in the first column

### Outcomes

#### Implementation

Rheumatologists and IHPs recognized team-based care as a highly acceptable and appropriate approach to provide rheumatological care. Patients valued the perceived better access to care (short waiting time to initial appointment and availability of health professionals to respond to patient queries between visits) and comprehensive care, particularly delivered under one roof by health professionals who communicate with one another with awareness of medical history and care needs. The team enhanced equitable care by servicing healthcare needs for individuals with a breadth of conditions, health literacy levels, and from different geographic regions and socioeconomic backgrounds through adaptable care elements (such as tailoring patient counseling or follow-up plans based on patient needs). Sustainability is evidenced by longevity of the program, the protocolization of workflows, and ongoing beneficial impact on patient care. The program maintained fidelity to the vision of interdisciplinary, comprehensive, and accessible rheumatology care.

#### Service

The team-based model was perceived to achieve increased access to timely specialty care. The personalization of care delivery facilitated equitable access to care, ensuring that all patients have access to high-quality rheumatology and IHP services, regardless of demographic or personal circumstances.

#### Patient

In the short term, patients and health professionals perceived that the team-based care improved the management of rheumatic disease through improved patient knowledge, sufficient clinical support, and, in turn, treatment adherence. In the longer term, these outcomes were perceived to lead to improved rheumatic disease control leading to improved symptoms, function, and decreased risk of disease complications. Care satisfaction within a team-based model was perceived by patients and health professionals to be high.

### Implementation Research Logic Model and mechanisms of action

**Fig. 2:**
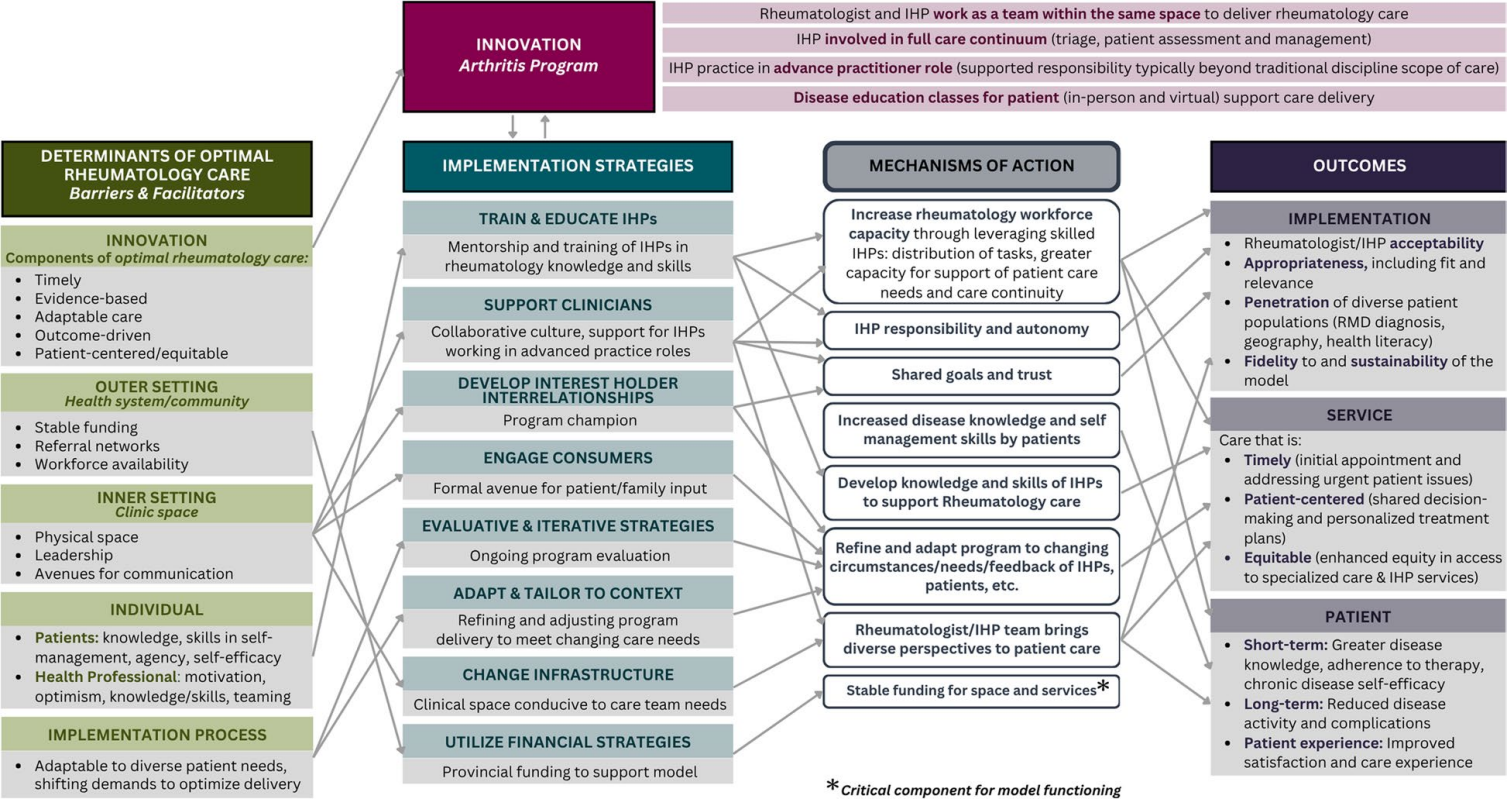
CArE logic model showing determinants, interventions, mechanisms, and resulting implementation, service, and patient outcomes

We present an IRLM of team-based rheumatology care at CArE in Fig. 2 that illustrates key mechanisms of action linking determinants to the innovation, implementation strategies, and outcomes. Key mechanisms of action are also specified in *italics*, below.

### Pathways contributing to implementation outcomes

IHP training and mentorship by rheumatologists facilitate development of advanced skills that IHPs need to thrive in an advanced-practice role *(Increase rheumatology workforce capacity)*. The increased autonomy to IHPs in the care they provide lead to greater personal satisfaction and re-investment into patient care (*IHP responsibility and autonomy)*. For rheumatologists and IHPs, witnessing the impact on patient care (both increase in workforce to see more patients and increased breadth of care provided to enhance quality of care) increases their confidence in and acceptability with this care model and reinforces their commitment to receiving (IHPs) and delivering (rheumatologists) ongoing training and mentorship. *Shared goals and trust* within the team support development of high-quality formal and informal relationships among the care team, which also increases acceptability. Patients re-iterated health professionals’ perceived acceptability, fit, and relevance of the model for its ability to provide expedited access to high-quality care.

### Pathways contributing to service outcomes

IHPs working alongside rheumatologists increase the skilled rheumatology workforce and capacity for patient visits through distribution of responsibilities *(Increased rheumatology workforce capacity)*. This distribution means greater availability to see patients and respond to concerns (e.g., accommodate urgent issues), expediting receipt of care services. While IHPs are trained in rheumatology-specific patient assessments, including history and physical exam *(Develop knowledge and skills of IHPs to support Rheumatology care)*, they also leverage their additional skills from their primary discipline (e.g., occupational therapy, pharmacy) to meet comprehensive care needs like rehabilitation or medication counselling *(Rheumatologist/IHP team bring diverse perspectives to patient care)*. The added service delivery within one team allows for a greater number of needs to be addressed within one clinic and set of providers, enhancing care coordination and integration. The provision of additional patient education sessions, and options for virtual and/or in-person delivery, based on IHP and patient feedback allows for enhanced disease knowledge, where applicable *(Refine and adapt program to changing circumstances/needs/feedback of IHPs, patients *etc*.).*

### Pathways contributing to patient outcomes

The timely and comprehensive care that the team-based model facilitates was perceived to increase patient disease knowledge and self-efficacy. Greater self-management skills result in knowledge of when and how to act if symptoms changed, and how the team can support the individuals’ health goals (*Increased disease knowledge and self-management skills by patients)*. The ability to provide frequent assessments when needed ensures proactive intervention, allowing the team to address evolving patient needs or complications quickly. The interdisciplinary team approach (*Rheumatologist/IHP team bring diverse perspectives to patient care)* brings diverse perspectives to patient care, which was seen to foster comprehensive, accessible, and high-satisfaction care. The combination of comprehensive and accessible care led to high care satisfaction. Patient outcomes are also achieved through enhanced continuity of care (resulting from increased rheumatology workforce capacity). This includes interpersonal/relationship continuity (fostering strong, trust-based relationships between team members and patients), and management continuity (consistent and coherent management of a patient's condition across different team members, ensuring treatment plans are aligned and adjusted as needed, avoiding duplication or gaps in care).

Overall, the mechanism of *Stable funding for space and services* was perceived to be a critical component for model functioning, which would impact all outcomes.

## Discussion

We used case study methodology to construct the program theory of a long-running interdisciplinary team-based model of rheumatology care. Through an IRLM, we have outlined key innovation components and associated implementation strategies and how they link to care determinants and outcomes. While interdisciplinary care is touted for its potential to improve care and outcomes for people living with chronic disease [18, 39], there is currently little literature around how to operationalize its implementation within community rheumatology practices. This understanding of the critical components of team-based care and how they act to achieve their effects, while of one model in one setting, provides a critical foundation of knowledge to contribute to optimal implementation, spread, scale, and sustainability of team-based care models.

We identified several key innovation components and implementation strategies at CArE that overcome barriers or enhanced enablers of high-quality rheumatology care to deliver timely, patient-centered care, and ultimately optimize patient outcomes. Based on our results, those implementing interdisciplinary teams at different sites may want to consider the following: First, formal and informal training for IHPs is critical to equip them with the knowledge of rheumatic diseases and necessary skills – particularly in physical examinations – enabling them to thrive in the rheumatology environment. Second, an investment into a culture of shared values and trust is important to optimize collaborative potential and output. Third, the physical space and other infrastructure should be set up mindfully to facilitate collaboration, including use of a shared medical record system, and standardized outcome metrics to provide a shared language. Fourth, a designated champion can cultivate a shared team mission, provide strong leadership, accountability, and supportive policies to sustain the model and program refinements as needed to support best care teams. Fifth, use of evaluative and iterative strategies can formally assess patient and provider needs, and facilitate program refinements. While considering these points, the functioning of the model can only be possible when stable funding is secured to support the IHP salaries and added infrastructure for IHPs and rheumatologists to work collaboratively. In this case example, funding was provided through the government insurer, via a provincial health agency. Stable funding should be viewed as an essential element for this model to operate, which highlights the need for cost-effectiveness research. While limited research exists in rheumatology, our findings are broadly consistent with research from primary care, where incorporating specific training [40], organization of clinic space [41], culture of shared values and principles [42], the need for champions [43], sources of external financial support [43, 44], and support for the logistics of implementation of primary care practice interdisciplinary teams [43] have been found to be important for optimal patient care. This overlaps with evidence relating to Wagner’s Chronic Care model [45] that emphasizes emphasizing proactive, planned, and patient-centered care, and with learnings from team effectiveness research, where teams are best understood as evolving systems, with effectiveness emerging from the interplay of compositional, structural, and mediating factors, along with contextual influences [46].

The increasing prevalence of RMDs has resulted in a demand for rheumatology care that surpasses the supply of rheumatologists, necessitating innovative care models to provide timely and effective treatment. Across Canada, physicians and policymakers are recognizing the value of interdisciplinary team-based care as a way to enhance integrated care for individuals with chronic disease, and widely believe team-based care can improve efficiency and quality [47] in the face of physician workforce shortages [48]. Despite increasing interest in physicians adopting an interdisciplinary practice, little has been published related to community outpatient rheumatology care. High-quality implementation research can fill this gap. Case studies are used within implementation research to identify and disseminate how the delivery of a complex intervention is achieved, why the implementation strategies produce change, under what circumstances (i.e., the impact of context), and to whom the implementation strategies produce change on [49]. Future research and practical application of this work can focus on how our IRLM can be operationalized by other teams to support spread and scale of this model.

Our study has multiple strengths. To our knowledge, this is the first study investigating the program theory of interdisciplinary team-based rheumatology care. We comprehensively collected data prospectively from multiple sources to allow for robust data triangulation and increased credibility. The IRLM is a widely used framework for describing a program theory, providing a shared language upon which others can build and compare. Our study also has limitations. The volume of data made it difficult to present the full depth of the results. We did not sample patients who exclusively received care from IHPs. The experiences and satisfaction of these patients may differ, however, their service and patient outcomes (and mechanisms of action) are likely distinct from those who require ongoing rheumatology support, which was our focus. The IRLM produces a linear depiction of relationships, however these relationships are rarely linear, and our approach may inadvertently convey simplistic views of complex processes. In this study, we applied the IOF conceptually to guide data collection and analysis; however, we did not collect quantitative, measurable implementation outcomes (e.g., adoption rates, penetration, or cost). Instead, our assessment focused on interest holders’ perceptions of key proximal implementation constructs (e.g., acceptability, appropriateness, feasibility, and fidelity). Future studies should incorporate quantitative measures of implementation, service and patient outcomes to strengthen evaluative rigour. Although we aimed to explore the model’s ability to deliver equitable care and reach all appropriate patients with RMDs (penetration), this could not be determined with the available data and will be explored further in future studies. Our research involved team-based care at one site within one health care system which may limit transferability of findings to other contexts. Further research should incorporate data from different settings to build an overarching logic model of team-based care.

## Conclusions

In conclusion, using case study methodology, we have elucidated the program theory of an interdisciplinary model of rheumatology care. Through identifying key innovation components and implementation strategies that overcome barriers and enhances enablers of optimal care and drive positive outcomes, this research provides a detailed and action-oriented foundation for health professionals and policy makers looking to implement team-based care within rheumatology. Future research is needed to explore the program theory of team-based care models at other sites to develop a comprehensive logic model (with adaptable innovation components) that may be widely applicable to guide spread and scale.

## Supplementary Information

Additional file 1.COREQ (COnsolidated criteria for REporting Qualitative research) Checklist

Additional file 2. Guide for patient interviews

Additional file 3. Guide for interviews of healthcare professionals and administrative staff

Additional file 4. Guide for clinic observations

## Data Availability

The data that support the findings of this study are not openly available due to reasons of sensitivity and may be available from the corresponding author upon reasonable request. Data are located in controlled access online data storage hosted by the University of Toronto.

## Abbreviations

CArE: Centre of Arthritis Excellence
CFIR: Consolidated Framework for Implementation Research
COREQ: Consolidated Criteria for Reporting Qualitative Research
ERIC: Expert Recommendations for Implementing Change
IHP: Interdisciplinary health professionals
IOF: Implementation Outcomes Framework
IRLM: Implementation Research Logic Model
RMD: Rheumatic and musculoskeletal disorders
THINC: Transforming Health with Integrated Care

## References

[1] Widdifield J, Paterson JM, Bernatsky S, Tu K, Thorne JC, Ahluwalia V, et al. The rising burden of rheumatoid arthritis surpasses rheumatology supply in Ontario. Can J Public Health. 2013;104(7):e450–5. 24495819 10.17269/cjph.104.4115 PMC6973680

[2] Eder L, Widdifield J, Rosen CF, Cook R, Lee KA, Alhusayen R, et al. Trends in the Prevalence and Incidence of Psoriasis and Psoriatic Arthritis in Ontario, Canada: A Population-Based Study. Arthritis Care Res. 2019;71(8):1084–91. 10.1002/acr.23743 30171803

[3] Widdifield J, Jaakkimainen L, Gatley J, Hawker G, Lix L, Bernatsky S, et al. Validation of Canadian health administrative data case definitions for estimating incidence and prevalence of Osteoarthritis. Osteoarthritis Cartil Open. 2020;2(4):100115. 10.1016/j.ocarto.2020.100115 PMC9718092 36474895

[4] Kulhawy-Wibe SC, Widdifield J, Lee JJY, Thorne JC, Yacyshyn EA, Batthish M, et al. Results from the 2020 Canadian Rheumatology Association’s workforce and wellness survey. J Rheumatol. 2022;49(6):635–43. 35105708 10.3899/jrheum.210990

[5] Jaakkimainen L, Glazier R, Barnsley J, Salkeld E, Lu H, Tu K. Waiting to see the specialist: patient and provider characteristics of wait times from primary to specialty care. BMC Fam Pract. 2014;15:16. 24460619 10.1186/1471-2296-15-16 PMC3912928

[6] Hurst NP, Lambert CM, Forbes J, Lochhead A, Major K, Lock P. Does waiting matter? A randomized controlled trial of new non-urgent rheumatology out-patient referrals. Rheumatology. 2000;39(4):369–76. 10817768 10.1093/rheumatology/39.4.369

[7] Isaacs JD. Decade in review-clinical rheumatology: 10 years of therapeutic advances in the rheumatic diseases. Nat Rev Rheumatol. 2015;11(11):628–30. 26481436 10.1038/nrrheum.2015.138

[8] Badley EM, Davis AM. Meeting the challenge of the ageing of the population: issues in access to specialist care for arthritis. Best Pract Res Clin Rheumatol. 2012;26(5):599–609. 23218425 10.1016/j.berh.2012.09.002

[9] Pollard LC, Graves H, Scott DL, Kingsley GH, Lempp H. Perceived barriers to integrated care in rheumatoid arthritis: views of recipients and providers of care in an inner-city setting. BMC Musculoskelet Disord. 2011;12:19. 21241497 10.1186/1471-2474-12-19 PMC3031274

[10] Harmsen S, Nabuurs JA, Lehman de Lehnsfeld LF, van der Meij MG, Broerse JEW, Pittens C. Mapping the complex everyday challenges and needs of people with rheumatic disease and their surroundings using a multi-actor approach. Musculoskeletal Care. 2022;20(4):873–91. 35478485 10.1002/msc.1639 PMC10084345

[11] Barber CEH, Lacaille D, Hall M, Bohm V, Li LC, Barnabe C, et al. Priorities for High-quality Care in Rheumatoid Arthritis: Results of Patient, Health Professional, and Policy Maker Perspectives. J Rheumatol. 2021;48(4):486–94. 33191276 10.3899/jrheum.201044

[12] Nancarrow SA, Booth A, Ariss S, Smith T, Enderby P, Roots A. Ten principles of good interdisciplinary team work. Hum Resour Health. 2013;11(1):19. 23663329 10.1186/1478-4491-11-19 PMC3662612

[13] Passalent L, Hawke C, Lawson DO, Omar A, Alnaqbi KA, Wallis D, et al. Advancing Early Identification of Axial Spondyloarthritis: An Interobserver Comparison of Extended Role Practitioners and Rheumatologists. J Rheumatol. 2020;47(4):524–30. 31043543 10.3899/jrheum.180787

[14] Bearne LM, Byrne AM, Segrave H, White CM. Multidisciplinary team care for people with rheumatoid arthritis: a systematic review and meta-analysis. Rheumatol Int. 2016;36(3):311–24. 26563338 10.1007/s00296-015-3380-4

[15] Garner S, Lopatina E, Rankin JA, Marshall DA. Nurse-led care for patients with rheumatoid arthritis: a systematic review of the effect on quality of care. J Rheumatol. 2017;44(6):757–65. 28202747 10.3899/jrheum.160535

[16] Hall J, Julia Kaal K, Lee J, Duncan R, Tsao N, Harrison M. Patient satisfaction and costs of multidisciplinary models of care in rheumatology: a review of the recent literature. Curr Rheumatol Rep. 2018;20(4):19. 29550993 10.1007/s11926-018-0727-3

[17] Apantaku G, Aguiar M, Kaal KJ, Munro S, Teo M, Harrison M. Understanding multidisciplinary care for people with rheumatic disease in British Columbia, Canada, through patients, nurses and physicians voices: a qualitative policy evaluation. BMC Health Serv Res. 2021;21(1):1148. 34688296 10.1186/s12913-021-07138-0 PMC8542329

[18] Position Statements on Priority Areas to Support the Sustainability of the Canadian Rheumatology Workforce Canadian Rheumatology Association. 2022. Available from: https://rheum.ca/wp-content/uploads/2023/02/CRA-Workforce-Position-Paper-Summary-EN-1.pdf.

[19] Greenhalgh T, Papoutsi C. Spreading and scaling up innovation and improvement. BMJ (Clinical research ed). 2019;365:l2068. 31076440 10.1136/bmj.l2068 PMC6519511

[20] Skivington K, Matthews L, Simpson SA, Craig P, Baird J, Blazeby JM, et al. A new framework for developing and evaluating complex interventions: update of Medical Research Council guidance. BMJ. 2021;374:n2061. 34593508 10.1136/bmj.n2061 PMC8482308

[21] Harris JA, Bykerk VP, Hitchon CA, Keystone EC, Thorne JC, Boire G, et al. Determining best practices in early rheumatoid arthritis by comparing differences in treatment at sites in the Canadian Early Arthritis Cohort. J Rheumatol. 2013;40(11):1823–30. 24037554 10.3899/jrheum.121316

[22] Widdifield J, Bernatsky S, Pope JE, Kuriya B, Barber CEH, Eder L, et al. Evaluation of Rheumatology Workforce Supply Changes in Ontario, Canada, from 2000 to 2030. Healthc Policy. 2021;16(3):119–34. 33720829 10.12927/hcpol.2021.26428 PMC7957360

[23] Yin RK. Case Study Research and Applications : Design and Methods. 6th ed. Thousand Oaks: SAGE Publications, Incorporated; 2017.

[24] Czosnek L, Zopf EM, Cormie P, Rosenbaum S, Richards J, Rankin NM. Developing an implementation research logic model: using a multiple case study design to establish a worked exemplar. Implement Sci Commun. 2022;3(1):90. 35974402 10.1186/s43058-022-00337-8 PMC9382723

[25] McLaughlin JA, Jordan GB. Logic models: a tool for telling your programs performance story. Eval Program Plann. 1999;22(1):65–72.

[26] Creswell JW, Poth CN. Qualitative inquiry & research design : choosing among five approaches. Fourth edition. ed. Thousand Oaks, California: SAGE; 2018.

[27] Crowe S, Cresswell K, Robertson A, Huby G, Avery A, Sheikh A. The case study approach. BMC Med Res Methodol. 2011;11:100. 21707982 10.1186/1471-2288-11-100 PMC3141799

[28] Fletcher A, Jamal F, Moore G, Evans RE, Murphy S, Bonell C. Realist complex intervention science: applying realist principles across all phases of the Medical Research Council framework for developing and evaluating complex interventions. Evaluation. 2016;22(3):286–303. 27478401 10.1177/1356389016652743 PMC4946011

[29] Damschroder LJ, Reardon CM, Widerquist MAO, Lowery J. The updated consolidated framework for implementation research based on user feedback. Implement Sci. 2022;17(1):75. 36309746 10.1186/s13012-022-01245-0 PMC9617234

[30] Powell BJ, Waltz TJ, Chinman MJ, Damschroder LJ, Smith JL, Matthieu MM, et al. A refined compilation of implementation strategies: results from the Expert Recommendations for Implementing Change (ERIC) project. Implement Sci. 2015;10(1):21. 25889199 10.1186/s13012-015-0209-1 PMC4328074

[31] Proctor EK, Powell BJ, McMillen JC. Implementation strategies: recommendations for specifying and reporting. Implement Sci. 2013;8(1):139. 24289295 10.1186/1748-5908-8-139 PMC3882890

[32] Waltz TJ, Powell BJ, Matthieu MM, Damschroder LJ, Chinman MJ, Smith JL, et al. Use of concept mapping to characterize relationships among implementation strategies and assess their feasibility and importance: results from the Expert Recommendations for Implementing Change (ERIC) study. Implement Sci. 2015;10(1):109. 26249843 10.1186/s13012-015-0295-0 PMC4527340

[33] Proctor E, Silmere H, Raghavan R, Hovmand P, Aarons G, Bunger A, et al. Outcomes for Implementation Research: Conceptual Distinctions, Measurement Challenges, and Research Agenda. Adm Policy Ment Health. 2011;38(2):65–76. 20957426 10.1007/s10488-010-0319-7 PMC3068522

[34] Six Domains of Healthcare Quality. Available from: https://www.ahrq.gov/talkingquality/measures/six-domains.html.

[35] Proctor EK, Landsverk J, Aarons G, Chambers D, Glisson C, Mittman B. Implementation research in mental health services: an emerging science with conceptual, methodological, and training challenges. Adm Policy Ment Health. 2009;36(1):24–34. 19104929 10.1007/s10488-008-0197-4 PMC3808121

[36] Carter N, Bryant-Lukosius D, DiCenso A, Blythe J, Neville AJ. The use of triangulation in qualitative research. Oncol Nurs Forum. 2014;41(5):545–7. 25158659 10.1188/14.ONF.545-547

[37] Gale NK, Heath G, Cameron E, Rashid S, Redwood S. Using the framework method for the analysis of qualitative data in multi-disciplinary health research. BMC Med Res Methodol. 2013;13(1):117. 24047204 10.1186/1471-2288-13-117 PMC3848812

[38] Smith JD, Li DH, Rafferty MR. The implementation research logic model: a method for planning, executing, reporting, and synthesizing implementation projects. Implement Sci. 2020;15(1):84. 32988389 10.1186/s13012-020-01041-8 PMC7523057

[39] Wagner EH. The role of patient care teams in chronic disease management. BMJ. 2000;320(7234):569–72. 10688568 10.1136/bmj.320.7234.569 PMC1117605

[40] Miller R, Scherpbier N, van Amsterdam L, Guedes V, Pype P. Inter-professional education and primary care: EFPC position paper. Prim Health Care Res Dev. 2019;20:e138. 31581968 10.1017/S1463423619000653 PMC6784359

[41] Ryan BL, Brown JB, Thorpe C. Moving from space to place: Reimagining the challenges of physical space in primary health care teams in Ontario. Can Fam Physician. 2019;65(9):e405–10. 31515328 PMC6741796

[42] Mayer RC, Davis JH. The effect of the performance appraisal system on trust for management: A field quasi-experiment. J Appl Psych. 1999;84(1):123.

[43] Barker KK, Bosco C, Oandasan IF. Factors in implementing interprofessional education and collaborative practice initiatives: findings from key informant interviews. J Interprof Care. 2005;19(Suppl 1):166–76. 16096153 10.1080/13561820500082974

[44] Freund T, Everett C, Griffiths P, Hudon C, Naccarella L, Laurant M. Skill mix, roles and remuneration in the primary care workforce: who are the healthcare professionals in the primary care teams across the world? Int J Nurs Stud. 2015;52(3):727–43. 25577306 10.1016/j.ijnurstu.2014.11.014

[45] Wagner EH. Chronic disease management: what will it take to improve care for chronic illness? Eff Clin Pract. 1998;1(1):2–4. 10345255

[46] Mathieu JE, Gallagher PT, Domingo MA, Klock EA. Embracing Complexity: Reviewing the Past Decade of Team Effectiveness Research. Ann Rev Org Psychol Organ Behav. 2019;2019(6):17–46.

[47] Pascucci D, Sassano M, Nurchis MC, Cicconi M, Acampora A, Park D, et al. Impact of interprofessional collaboration on chronic disease management: findings from a systematic review of clinical trial and meta-analysis. Health Policy. 2021;125(2):191–202. 33388157 10.1016/j.healthpol.2020.12.006

[48] Hogg W, Lemelin J, Dahrouge S, Liddy C, Armstrong CD, Legault F, et al. Randomized controlled trial of Anticipatory and Preventive multidisciplinary Team Care For complex patients in a community-based primary care setting. Can Fam Physician. 2009;55(12):e76–85. 20008582 PMC2793206

[49] Beecroft B, Sturke R, Neta G, Ramaswamy R. The, “case” for case studies: why we need high-quality examples of global implementation research. Implement Sci Commun. 2022;3(1):15. 35168672 10.1186/s43058-021-00227-5 PMC8848686

