# Supplementary material for: Constructing the program theory: an implementation science approach to understanding a successful interdisciplinary team-based model of rheumatology care
Source: Implement Sci Commun. 2026 Feb 6;7:45. doi: 10.1186/s43058-026-00870-w (PMC12973881; doi:10.1186/s43058-026-00870-w)
Supplement: Supplementary file 2 — Additional file 2. Guide for patient interviews [file 43058_2026_870_MOESM2_ESM.pdf]

ADDITIONAL FILE 1 – Interview Guide for Patients

Thank you for agreeing to speak with me today. I'm going to begin the interview by asking you a few questions in order to get to know you a bit better and to learn about your experiences living with a rheumatic disease and your interactions with the health care system. Since we are trying to better understand team-based models of rheumatology care, where rheumatology care is delivered by multiple different health professionals, I'll then ask you more about your experiences and perspectives about the Arthritis Program that also goes by the name Center for Arthritis Excellence, or CArE. We really want to hear from people like yourself who have received care at the Arthritis Program. These questions serve as a guide only. We encourage you to talk about any aspect of the topic you wish. There are no right or wrong answers to these questions. Questions will start very broadly and I'll ask some more specific questions towards the very end of the interview.

We will be audio recording the interview in order to capture all the details of our conversation. This is a purely voluntary activity, so remember that you may end the interview at any time. If you need to take a break, please let me know and we can do so. Also, if you feel uncomfortable with any question, tell me and we can skip it. No personal information about you will be shared with anyone outside of the study team. No identifying information will be shared in any reports or publications. While we might use direct quotes in these reports, they will only be attributed more generally to someone who is a "patient", etc. All the information you provide will be kept strictly confidential. We hope you will feel able to speak freely as we genuinely want to hear your perspectives.

If a question does not make sense, let me know so I can ask it more clearly. Take as much time as you like to answer the questions.

Do you have any questions before we begin?

(and if not, start recording)….

1. To begin, can you please tell me about how your experience living with a rheumatic disease began?

Probes:
   a. When and where were you diagnosed?

2. How did you get connected to the Arthritis program?
3. What has your experience been like with the Arthritis program?

Probes:
   a. What is it like to receive care through the Arthritis program?

As we are trying to understand the full patient journey through the Arthritis Program, the next few questions go into detail about your experience with the program.

4. Can you talk me through how you book an appointment?
    a. Can you describe the process of reaching the clinic if you have an urgent health issue to discuss? How long would it typically take to be seen for an urgent issue?

5. Multiple different types of healthcare professionals work at the Arthritis Program. During a routine follow up, do you normally see the rheumatologist only or do you typically see other healthcare professional (nurse, physiotherapist etc.)? *[If sees other health care professionals, probe: Who have you seen as part of your care?]*

6. Can you talk me through a typical rheumatology follow-up appointment?

   Probes:
    a. How long is the visit?
    b. Do you discuss your goals of care? If yes, can you provide an example?
    c. What is the communication like? Do you feel you are being heard? If yes, what tells you this?
    d. Tell me about the quality of the care you receive at the Arthritis Program.
    e. Do you feel the care is tailored to your specific needs? If yes, can you provide an example?
    f. Is there anything you would change about how your care is delivered?
    g. Have you been given any resources during or after your visit?
        i. If yes, were they useful?
        ii. If no, did you want any resources?
    h. What's it been like to access and take medications for your rheumatic condition? Have you had any challenges? (Prompt: biologic access, drug monitoring). What kind of support, if any, around medications is provided at the Arthritis Program?

7. Can you talk me through what typically happens after your visit? Prompt: making follow-up care arrangements, any xrays or lab tests you need to get done?

8. What's the process like for communicating with the Arthritis Program between visits, if for example, an urgent issue comes up? Do you feel your needs are met in terms of access to care when you need it? Why/why not?

9. Outside of a rheumatologist visit, have you ever scheduled an appointment with another health professional at the Arthritis Program (e.g., pharmacist, physiotherapist, occupational therapist, social worker?). If yes, can you talk me through what that looked like [for each healthcare professional], including the reason for the visit and what your care was like?

   Probes:
    a. How long is the visit?
    b. Do you discuss your goals of care? If yes, can you provide an example?
    c. What is the communication like? Do you feel you are being heard? If yes, what tells you this?
    d. Tell me about the quality of the care you receive at the Arthritis Program.

e. Do you feel the care is tailored to your specific needs? If yes, can you provide an example?
f. Is there anything you would change about how your care is delivered?
g. Have you been given any resources during or after your visit?
    i. If yes, were they useful?
    ii. If no, did you want any resources?

10. Do you see any healthcare professionals for your rheumatic condition outside of the Arthritis Program? (If yes, who do you see and for what aspect of your care? Who referred you to them?)

11. What do you think about the alliance or therapeutic relationships between those providing care (the rheumatologist, the other healthcare professionals, the administrative staff etc.)?
    Prompt: good communication? Respectful?

12. What do you think about the physical space of the clinical overall? What about the room where you have your visit?
    Prompt: Welcoming? Crowded?
    a. Did the physical space accommodate your specific needs? (e.g., accessible washrooms)
    b. Have you ever observed that the space is lacking some accommodations for others?

13. Have you ever attended a group-based program based at the Arthritis Program? If so, what was that like? If not, why not?
    Probes:
    a. How long is program (how many visits over how long)?
    b. Can you describe the quality of the care you received?
    c. Do you feel the care is tailored to your specific needs? If so, can you provide examples. If not, can you describe why?
    d. Is there anything you would change about the program itself, or the types of programs offered?
    e. Were you given any resources during or after the group program?
        i. If yes, were they useful?
        ii. If no, did you want any resources?

14. What do you think about the communication between the Arthritis Program and your family physician? What about with any other physicians or health professionals involved in your care? Prompts: timely? slow? accurate?

15. If a program similar to the Arthritis Program was to be set up somewhere else, what do you think are the key parts of this program that would be required that would support the best care for patients? Prompt: mix of healthcare professionals; physical space; location

Probe:

a. If the program were to start small, what is the first thing that would be needed? Prompt: what is the most important part of the care in the program?
b. What do you think should be added to this new clinic?

16. Is there any part of the current Arthritis Program that you do *not* think needs to be continued?
17. Are there any parts of the healthcare system that have made receiving care more difficult for you? If so, what made it difficult? Prompt: Coverage for medications, braces/splints, physical therapy etc.
18. Is there anything else you wish to say related to the care you receive at the Arthritis Program? Is there anything else you would like to mention that we didn't talk about today?
19. I would like to finish by asking about some details about you and your rheumatic disease. What is your:

- Age? (years)
- Self-identified gender?
- Highest level of education received (less than high school completion, high school completion, college or university, post graduate)
- Race/ethnicity that you identify most with?
- Location of residence? (City/town)
- Rheumatic disease diagnosis? (e.g., rheumatoid arthritis, psoriatic arthritis, systemic lupus erythematosus etc)
- How long have you lived with this rheumatic disease? (Years since diagnosis)
- How long have you been a patient of the Arthritis Program? (years)
- Do you take medication for your rheumatic disease treatment? (Name of medication(s) currently using for your rheumatic disease)
