# Supplementary material for: Constructing the program theory: an implementation science approach to understanding a successful interdisciplinary team-based model of rheumatology care
Source: Implement Sci Commun. 2026 Feb 6;7:45. doi: 10.1186/s43058-026-00870-w (PMC12973881; doi:10.1186/s43058-026-00870-w)
Supplement: Supplementary file 3 — Additional file 3. Guide for interviews of healthcare professionals and administrative staff [file 43058_2026_870_MOESM3_ESM.pdf]

ADDITIONAL FILE 2 – Interview Guide for Healthcare Professionals and Administrative Staff

Thank you for agreeing to speak with me today. We're in the first phase of a study that is trying to better understand team-based models of care in rheumatology and as part of this we really want to hear what things have been like at The Arthritis Program and the Center for Arthritis Excellence. For short, we're just going to call it the Arthritis Program, but we're referring to the model that rheumatology care is delivered as part of these entities.

I'm going to start the interview by asking you a few questions in order to get to know you and your roles a little bit more. Then I'm going to ask you about the perspective about the model at the Arthritis Program, and in particular your experiences working at the clinic and what you see as some of the key components that are essential for it to function.

I have a list of questions to go through that serve as a guide only. I encourage you to talk about any aspect of the topic you wish. There are no right or wrong answers to these questions.

I will be audio recording the interview, in order to capture all the details of our conversation. This is a purely voluntary activity, so remember that you may end the interview at any time. If you need to take a break, please let me know and we can do so. Also, if you feel uncomfortable with any question, tell me and we can skip it. No personal information about you will be shared and you will not be identified to your colleagues or patients. No identifying information will be shared in any reports or publications. While we might use direct quotes in such reports, they will only be attributed more generally to someone who is a "health professional". All the information provided by you will be kept strictly confidential and we hope you will feel able to speak freely as we genuinely want to hear your perspective.

If a question does not make sense, let me know so I can ask it more clearly. Take as much time as you like to answer the questions.

Do you have any questions before we begin?

(and if not, start ***recording****)….*

1. Can you please start by describing your role at the Arthritis Program? What does a typical day look like for you?

Probes:

a. What types of patients do you see?
b. How many patients do you see in a typical day? On a busy day?
c. How much time is allotted per patient?
d. How much time do you actually spend with each patient?

As you know, we are looking into details of how the Arthritis Program works, so the next few questions are about the goals of the program, and then we'll go through some details of the process for a patient.

2. If you were to describe the Arthritis Program to someone who knows nothing about it, what would you say?
3. What are the overall goals and priorities of the program?
   a. How does the program work towards meeting those goals?
   b. Do you think these goals are being met?
   c. Do you think there are any changes needed to the goals themselves?
   d. Do you think there are any changes needed in how the goals are being (or not being) met?
   e. Do you think these goals and priorities meet the needs of all patients? *[health equity perspective]*

Patient Journey/Innovation/Inner Setting

4. Can you talk me through the process for a patient in the program, starting from the first point of connection with the program (appointment request, referral etc.)?
   a. What is the triage process?
   b. What is a typical wait time? How are wait times monitored?
   c. What kind of information is collected before the first visit?
   d. What does a typical rheumatologist visit look like? (who do they see as part of the visit etc.) When might a patient schedule a specific visit with another healthcare professional (i.e., not a rheumatologist)? How does that process work?
   e. What happens after a visit? (follow-up process, given resources etc.)
5. Can you tell me about the group programs offered?
   a. How does a patient access the group program?
   b. What are the benefits of this approach?
   c. What are the challenges?
6. Now that we've gone through the details of this process, what do you think about the current process?
   a. Is care patient-centered?
   b. Is there enough consideration of delivery of equitable care?
   c. Are there any changes you think should be made to the process at this clinic? If so, what should change?
   d. If a new program were starting somewhere else, what would you recommend is done differently? The same?

Inner Context/People

7. Can you tell me about your experience with having multiple healthcare professions delivering care in this program?
    a. Who are the other healthcare professionals that you interact with/work together with? (*note: this might be different depending on when they worked at TAP/CaRE*)
    b. What are the benefits?
    c. What are the challenges?
    d. What personal characteristics do you think a healthcare professional needs to work effectively in this program?
    e. Do you think other similar programs should take the same approach? Why/why not?
    f. If another program were to start this approach, what do you think they should know? Is there anything that could be done differently? Should be kept the same?
8. What type of training is provided to new people when they join the team?
    a. Is this training enough? If not, what else is needed?
    b. What is done to make new members feel they are "part of the team"? Is this enough?

Health Equity

9. How do you think the program is doing at meeting the needs of all patients, considering geography, culture/ethnicity, education level, income, or other personal characteristics?
    a. Can you provide an example of how the clinic does, or does not, support patients from different geographical regions?
    b. Can you provide an example of how the clinic does, or does not, support patients from different cultures/ethnicities?
    c. How does the clinic accommodate different health literacy levels?
    d. Is there anything that should be done differently to meet these needs?
    e. If a new clinic were to be set up, what do you think should be done differently from the beginning to meet these needs? Kept the same?

Evaluation

10. How do you know if the program is meeting the needs of its patients? Prompt: Is there any formal evaluation?
    a. Do you think more evaluation is needed?
        a. If so, what should be measured? (i.e., patient satisfaction etc.)
    b. How will you know if you are meeting the needs of all patients, considering the topics we discussed around geography, ethnicity etc.

Adaptations & Sustainability

11. How does the program change to meet the evolving needs of its patients?

a. Are there changes in process (triage etc.)?
b. Are there changes in physical space?
c. Are there changes in team structure (adding a new HCP etc.)?

12. Do you feel comfortable to suggesting changes in how thing work in the program? Why/why not?
    a. Do you have an example of when you recommended a change in process? How did it go?
13. What changes do you think are needed now, or will be needed in the future, to keep the program going?

System level/Outer Context

14. For this project, we are looking into how a program similar to this could be set up elsewhere. What do you think we should consider if there were limited resources?
    a. Thinking optimistically, what would you like to see if there were unlimited resources?
    b. What are some of the barriers within the province and existing health care system relevant to care for people with rheumatic disease?
    c. How does the Arthritis Program overcome some of these barriers?
    d. What would help patients access rheumatology care in a more timely manner?
    e. How else could the healthcare system better support good quality rheumatology care?
15. Do you have anything else you wish to say related to the Arthritis Program or more generally around caring for people with rheumatic disease? Is there anything else you'd like to raise that we didn't talk about today?
16. I would like to finish with briefly asking a few demographic questions which we're asking all our participants. What is your:
    - Age: (years)
    - Gender:
    - Professional designation (PT, OT, rheum, RN etc):
    - Current employment status (full time/part time/not employed/retired):
    - Current employer/employment location? (CArE vs other [specify]):
    - Numbers of years working at TAP/CArE (specify years)
    - Number of years in professional practice (specify years)
