# Supplementary material for: Constructing the program theory: an implementation science approach to understanding a successful interdisciplinary team-based model of rheumatology care
Source: Implement Sci Commun. 2026 Feb 6;7:45. doi: 10.1186/s43058-026-00870-w (PMC12973881; doi:10.1186/s43058-026-00870-w)
Supplement: Supplementary file 4 — Additional file 4. Guide for clinic observations [file 43058_2026_870_MOESM4_ESM.pdf]

ADDITIONAL FILE 3 – Clinic Observation Guide

CFIR Constructs (determinants that might influence implementation)	Prompts
**INNOVATION (interdisciplinary model of care, clinical encounter)**	• Context (intake process, clinical programs)<br>• Advantage/Benefit (reason for referral)<br>• Complexity/Ease (duration of clinical encounters)<br>• Acceptability (patient satisfaction survey)<br>• Penetration (wait time)<br>• Adapt/Tailor
**INNER SETTING (CArE clinic)**	• Physical Space (waiting room, number and size of rooms, sufficient chairs for caregivers, set up of clinic room, etc.)<br>• Work Infrastructure (EMR, billing/fees)<br>• Relationships & Communication (EMR, telephone, face-to-face, memos posted)<br>• Culture (Equity, Patient-Centeredness) (language used when communicating with patients, patient population)<br>• Priorities (observed emphasis of care)<br>• Resources (information for patients, intake forms, EMR)<br>• Training/Education/Support (onboarding process, staff training, staff meetings)
**INDIVIDUALS (people working at or attending CArE)**	• Leadership (who is involved and their roles, support for team members, how team members interact)<br>• Healthcare Professionals (type and number, roles they take on [note - may not be typical for discipline], how they interact)<br>• Patients (journey, rheumatic condition, severity and duration of condition, area of Ontario they live in, level of engagement, preparedness, power and decision making, therapeutic alliance)<br>• Perspective (patient feedback, staff feedback)<br>• Acceptability (patient feedback, staff feedback)

CFIR Constructs (determinants that might influence implementation)	Prompts
**IMPLEMENTATION PROCESS (activities and strategies)**	• Reflecting & Evaluating (any mechanisms for soliciting patient feedback, staff feedback)<br>• Adaptability & Fidelity (how closely is same model followed vs individualized to specific patient needs e.g., PT involvement, pharmacy)
**OUTER SETTING (Ontario health system)**	• Partnerships & Communication (referrals to/from outside of CArE)<br>• Attitudes, Priorities, & Conditions (referral process)<br>• Performance-Measurement (any evidence of quality monitoring system)
